# Exercise Improves Host Response to Influenza Viral Infection in Obese and Non-Obese Mice through Different Mechanisms

**DOI:** 10.1371/journal.pone.0129713

**Published:** 2015-06-25

**Authors:** Kristi J. Warren, Molly M. Olson, Nicholas J. Thompson, Mackenzie L. Cahill, Todd A. Wyatt, Kyoungjin J. Yoon, Christina M. Loiacono, Marian L. Kohut

**Affiliations:** 1 Immunobiology Program, Iowa State University, Ames, IA, United States of America; 2 Department of Kinesiology, College of Human Sciences, Iowa State University, Ames, IA, United States of America; 3 VA Nebraska-Western Iowa Health Care System Research Service, Department of Veterans Affairs Medical Center, Omaha, NE, United States of America; 4 Pulmonary, Critical Care, Sleep & Allergy Division, University of Nebraska Medical Center, Nebraska Medical Center, Omaha, NE, United States of America; 5 National Veterinary Services Laboratories, USDA, APHIS, Ames, IA, United States of America; 6 Department of Veterinary Diagnostic and Production Animal Medicine, Iowa State University, Ames, IA, United States of America; St. Jude Children's Research Hospital, UNITED STATES

## Abstract

Obesity has been associated with greater severity of influenza virus infection and impaired host defense. Exercise may confer health benefits even when weight loss is not achieved, but it has not been determined if regular exercise improves immune defense against influenza A virus (IAV) in the obese condition. In this study, diet-induced obese mice and lean control mice exercised for eight weeks followed by influenza viral infection. Exercise reduced disease severity in both obese and non-obese mice, but the mechanisms differed. Exercise reversed the obesity-associated delay in bronchoalveolar-lavage (BAL) cell infiltration, restored BAL cytokine and chemokine production, and increased ciliary beat frequency and IFNα-related gene expression. In non-obese mice, exercise treatment reduced lung viral load, increased Type-I-IFN-related gene expression early during infection, but reduced BAL inflammatory cytokines and chemokines. In both obese and non-obese mice, exercise increased serum anti-influenza virus specific IgG2c antibody, increased CD8+ T cell percentage in BAL, and reduced TNFα by influenza viral NP-peptide-responding CD8+ T cells. Overall, the results suggest that exercise “restores” the immune response of obese mice to a phenotype similar to non-obese mice by improving the delay in immune activation. In contrast, in non-obese mice exercise treatment results in an early reduction in lung viral load and limited inflammatory response.

## Introduction

Obesity is a known risk factor for multiple disease states including metabolic disease, cardiovascular disease and types of cancer [1,2,3,4]. Studies suggest that obesity is correlated with an increased risk and severity of infectious disease of viral or bacterial origin [5,6]. In the recent 2009 H1N1 influenza epidemic, obesity was associated with increased hospitalization and infection severity [7,8,9]. Also suboptimal antibody responses to various vaccinations, including influenza vaccine, have been identified in overweight individuals [10,11,12]. These findings suggest that obesity may cause impaired immune responsiveness, yet the mechanisms responsible are currently being defined, and strategies to improve immune function in obese populations remain to be elucidated.

Previous studies have shown a poorer disease outcome to influenza A virus (IAV) infection in obese mice compared to non-obese mice [13,14,15,16]. In response to primary IAV infection, immune cell infiltration and cytokine/chemokine production (IFNα/β, TNFα, G-CSF, CXCL-10, MCP-1 and RANTES) were delayed or reduced in the lungs of obese mice [13,14,15,17]. Dendritic cell impairments have been implicated in the early loss of immune activation with subsequent effects on CD8+ T cell function. In addition, the primary CD8+ T cell response was delayed and reduced in comparison to non-obese controls, and reduced T cell memory and maintenance of memory T cells in obese mice after IAV challenge has been shown [18,19]. This memory response was less protective in obese mice as 25% mortality occurred upon secondary IAV challenge in comparison to no mortality in the non-obese mice. The existing literature generally demonstrates that obesity is associated with delays in innate immune activation, which may contribute to the development of a suboptimal adaptive immune response.

Although awareness has grown with respect to the health consequences of obesity, the development of effective strategies to treat the condition has been an ongoing challenge. The results from several studies show some promise by demonstrating that morbidity and mortality may be reduced if the health practice of regular exercise is maintained, even under conditions in which individuals remain overweight. In fact, overweight individuals that exercise regularly may have equivalent or *reduced* mortality compared to normal weight individuals that do not exercise. Long term cohort studies showed that individuals that demonstrated greater aerobic fitness, even with a body mass index (BMI) classified as overweight (BMI = 25–30 kg/m^2^), have reduced mortality from multiple disease conditions (e.g., metabolic or cardiovascular disorders) relative to those of poorer fitness level [20,21,22,23]. However, it is not known whether exercise may ameliorate the negative effect of obesity on infectious disease outcome. A major objective of this study was to establish the extent to which moderate exercise may improve host defense against influenza A viral infection in the high fat diet-induced obese mouse model. This model of obesity was used to parallel the human condition in which diet contributes to obesity and the maintenance of ideal body weight remains a significant challenge. In this study, an exercise program commenced one week following the start of a high fat diet treatment to allow sufficient time for the development of the obese state, but prior to development of diabetic-associated metabolic changes. This approach provided a means of evaluating whether exercise in an overweight condition accompanied by a suboptimal diet could confer protection from infectious disease, thus mirroring a situation common to a significant portion of the human population.

Another objective of this study was to identify host defense responses that were altered by exercise, and determine whether these mechanisms were the same in the obese and non-obese condition. In previous studies of non-obese mice, moderate exercise improved host defense against IAV or other respiratory infection in non-obese mice, yet exhaustive exercise increased the severity of infectious disease [24,25]. Whether this same benefit occurs in the obese condition has not been determined, and the mechanisms responsible for such an effect remain to be clearly established, although some evidence indicates that exercise may have a local “anti-inflammatory” effect during acute infection [26]. Exercise affects energy balance as well as leptin response. It has been shown that other factors involving energy intake, namely caloric restriction, can actually impair host defense against IAV infection [27]. Other nutritional strategies such as the administration of fish oil to limit inflammation had a detrimental effect on host resistance to influenza infection resulting in increased lung viral load [28]. Based on these studies, it is possible that exercise may improve immune defense against IAV infection in the obese, yet it also remains possible that through energy alterations or other metabolic effects, exercise may not serve a protective role. The purpose of this study was to determine whether exercise serves any benefit in obese populations during influenza viral infection, and to establish the mechanisms that may be responsible for such an effect. Briefly, the results showed that exercise improved host immunity to IAV infection in both obese and non-obese mice, but largely through different mechanisms. In obese mice, exercise reversed the obesity-associated impairments in host immune defense. In contrast, in non-obese mice exercise contributed to an activation of early anti-viral defenses resulting in reduced viral load. These findings have important public health implications in reducing the severity of infectious disease, and also provide further insight into “immuno-regulatory” effects of exercise on the respiratory tract.

## Materials and Methods

### Mice

This study was performed in accordance with the recommendations in the Guide for the Care and Use of Laboratory Animals of the National Institutes of Health. The protocol was approved by the Institutional Animal Care and Use Committee of Iowa State University (Protocol Number: 11-11-7256-M). Six-week old, male C57BL/6 mice were purchased from Jackson laboratories (Bar Harbor, ME). Mice were housed on a 12:12 hour light:dark cycle.

### Diet

After a seven day acclimation period in the animal facility, mice were randomly assigned to a 10% kcal from fat diet (TestDiet, Richmond IN, Formula 58Y2) to maintain the lean phenotype (Non-ob); or mice were placed on a matched 60% kcal from fat diet (TestDiet, Richmond IN, Formula 58Y1) to achieve the diet-induced obese phenotype (Ob). Mice were fed *ad libitum*. The energy composition of the Formula 58Y2 diet was 18.3% protein, 10.2% fat, and 71.5% carbohydrate, with the primary source of protein as casein, the primary sources of fat as lard and soybean oil, and the primary sources of carbohydrates as sucrose, dextrin and maltodextrin. The Formula 58Y1 diet contained 18.1% protein (primary source casein), 61.6% fat (primary source lard with soybean oil added), 20.3% carbohydrate (primary sources as maltodextrin and sucrose). These diet formulations have been used in other research studies and the high fat diet results in typical alterations of glucose and insulin resistance associated with the obese condition [29,30]. Body weight and food weight were measured weekly over the course of the study. In order to maintain the non-obese or obese phenotype achieved after nine weeks of diet treatment, mice remained on their respective diet until the conclusion of the study.

### Exercise Protocol

After one week of diet treatment, half of the mice on each diet were randomly selected to acclimate to a motorized treadmill to undergo an eight-week training protocol, previously described [26,31]. Briefly, mice were randomly divided into four groups: non-obese non-exercise (Non-ob NO-EX); non-obese exercise (Non-ob EX); obese non-exercise (Ob NO-EX); and obese exercise (Ob EX). Non-exercised groups were exposed to similar handling and treadmill noise as exercised mice. All randomly selected mice adapted well to daily treadmill running and gradually the exercise speed and duration of each session was increased from 12 m/minute for 10 minutes until mice were running 45 minutes/day, five days a week at 18 m/minute. This intensity of exercise corresponds to 65–80% VO_2_ max, an intensity considered to be moderate in humans [32,33,34]. At the time which exercise training began (after one week of diet treatment), mice were separated into individual cages and body weight was monitored on a weekly basis.

### Influenza Viral Infection

Twenty-four hours after the last exercise session mice were briefly anesthetized with aerosolized isoflurane and infected with Influenza A/PR/8/34 [5.58 X 10^2^ median chicken embryo infectious dose (EID50) per mouse in a 25 μl volume]. Non-infected control mice were administered 25 μl of sterile saline using the same anesthesia procedure. Mice were not exercised after infection as prolonged exercise after infection results in greater morbidity [24,35]. Body weight and food consumption were monitored daily through the infection portion of the study as a means of assessing illness severity in each of the groups of mice. Mice from each of the four treatment groups were euthanized at various time points post-infection (p.i.): day one, day three, day eight, and day eleven post-infection. At each euthanization time point, three to thirteen mice per group were included, based on power calculations for the immune parameter of interest using data from previous experiments.

### Dual-energy X-ray (DXA) Imaging

One week prior to infection a subset of 10 mice per group were randomly selected to undergo DXA imaging to determine body composition (total body fat % and abdominal fat %) after eight weeks of training and diet consumption. Mice were anesthetized with an intraperitoneal (i.p.) injection of ketamine:xylazine (90 mg/kg: 4.5 mg/kg) cocktail and then placed under heat lamps to maintain body temperature. Once mice were immobile they were quickly placed on the DXA imager for scanning (8 mice per scan) then returned to their cages for recovery. Mice were monitored until they recovered from anesthesia. Fat percentages were determined using the software for the DXA imager.

### Luminex assay for cytokine and chemokine detection

Bronchoalveolar lavage (BAL) fluids were collected and centrifuged at 160 x g to pellet BAL cells and remove debris from supernatants. BAL supernatants were stored at -80°C until time of testing. Cytokine and chemokine detection was performed using the Luminex 200 system (BIO-RAD) and corresponding plates. The Multiplex 32-plex or 9-plex (Millipore) kit, were used to detect cytokine and chemokines according to the manufacturer’s protocol.

### RNA Isolation, reverse transcription and polymerase chain reaction (PCR) for Type I interferon-related genes

The anatomical left lobe was collected for RNA isolation and purification using TRIzol Reagent (Invitrogen) and the RNeasy Micro Kit (Qiagen). Purified RNA was stored at -80°C until it was converted to cDNA using the RT^2^ First Strand Kit (Qiagen). 1,500 ng of cDNA was analyzed on a Mouse Interferon α, β Response or Inflammasomes RT^2^ Profiler PCR Array (SABiosciences). Detection was carried out on the BioRad iCycler and corresponding MyIQ Optical System Software Version 1.0. Cycling thresholds were normalized to housekeeping gene and fold-induction was determined by comparing infected to non-infected groups, or exercise relative to the non-exercise condition. For a subset of genes, protein levels were also measured (IL-1β, IFNα, CXCL10, CCL5 and CCL12).

### Viral titer quantification by quantitative RT-PCR

Viral load was determined by real-time quantitative reverse-transcription polymerase chain reaction (qRT-PCR) with TaqMan chemistry, previously described [26,31]. Using sequences deposited in GenBank (http://www.ncbi.nlm.nih.gov/Genbank/index.html) and the Influenza Sequence Database (http://www.flu.lanl.gov), viral-specific oligonucleotide primers and fluorescent probes were engineered. Target sequences were chosen based on highly conserved regions of the swine influenza viral nucleoprotein. The forward primer (SIVRTF: 5’-CGGACGAAAAGGCAACGA-3’) and reverse primer (SIVRTR: 5’-CTGCATTGTCTCCGAAGAAATAAG-3’) were synthesized by a commercial vendor (Integrated DNA Technologies). A TaqMan MGB probe with a 5’ reporter 6-carboxyfluorescein (FAM) and a 3’ nonfluorescent quencher (SIVRTP: 5’-6-FAM-CCGATCGTGCCTTC) was generated by a commercial vendor (Applied Biosystems). To conduct the assay, viral RNA was first extracted from a 50 μL aliquot of lung homogenate or BAL fluid, positive controls (H1N1 and H3N2 swine influenza viruses) of known viral concentrations (10^3^–10^8^ TCID50/mL), and negative control (elution buffer), using the Ambion MagMAX Viral RNA Isolation Kit (Applied Biosystems) and the KingFisher 96 magnetic particle processor (Thermo Scientific). qRT-PCR was performed using the QuantiTect Probe RT-PCR Kit (Qiagen) in a 20-μL reaction volume and 4 μL of extracted template. Primers were added at a final concentration of 0.4 μM each, and the probe was added at a final concentration of 0.2 μM. Amplification was carried out on the ABI 7900HT Sequence Detection System (Applied Biosystems). Samples with threshold cycle values of ≤ 35 Ct were considered positive. A standard curve was generated from the set of positive samples of known viral titer. The viral load (TCID_50_ equivalent/mL) was estimated by converting the threshold cycle value to a virus titer using the standard curve.

### Viral titer quantification by infectivity assay

BAL fluids from d1 p.i. and d3 p.i. infected mice were briefly centrifuged at 160 x g, then stored at -80°C until the infectivity assay could be completed. A549 cells were subcultured into 96-well cell culture plates (Costar) in DMEM medium containing 10% heat-inactivated FBS and penicillin-streptomycin (10 U/mL). Once cells reached 80–90% confluence, cell monolayers were washed two times with PBS then inoculated with 10-fold, serially diluted sample containing unknown amount of virus, stock virus as positive control or PBS alone as a negative control. Five replicates of each sample were inoculated into individual wells at dilutions ranging from 10^1^–10^8^. Stock A/PR/8/34 influenza virus was inoculated onto cells (diluted from 10^2^–10^9^). Cells were incubated with virus for 2 hours at 37°C, 5% CO_2_. Diluted virus was removed from the cells and post-inoculation media [DMEM supplemented with 2% BSA, Penicillin-Streptomycin (10 U/mL), and TPCK-treated trypsin (0.125 μg/mL)] was added to all the wells. The plates were incubated for 5 days and monitored for cytopathic effect. Median tissue culture infectious dose (TCID_50_) per mL was determined using the Reed-Muench method.

### Identification of Lung Cell Populations by Flow Cytometry

Cell populations were identified in bronchoalveolar lavage fluid by staining with the following antibodies: rat anti-mouse NK-1.1 APC-Cy 7, rat anti-mouse CD8α APC-Cy7, hamster anti-mouse CD11c PE-Cy7, rat anti-mouse CD3ε PerCP-Cy5.5, rat anti-mouse Ly6g PE (clone: 1A8), rat anti-mouse Gr-1 PE (clone: RB6-8C5), rat anti-mouse CD11b Alexa Fluor 700, rat anti-mouse MHC-II Alexa Fluor 700, rat anti-mouse mPDCA-1 APC, rat anti-mouse CD4 APC, and rat anti-mouse Ly6c APC (clone: HK1.4). Samples were analyzed on the FACS Canto and lung cell population were determined using FlowJo software (version 7.6). Day one and day three lung populations identified included neutrophils (SSC^hi^ CD11b^hi^ CD11c^lo^ Ly6g^hi^ Ly6c^hi^), NK cells (NK1.1^+^ CD3^-^), macrophages (CD11b^hi^ CD11c^-^ autofluorescence^hi^), plasmacytoid dendritic cells (CD11c^int^ mPDCA-1^+^), and TNFα/iNOS producing dendritic cells (CD11b^hi^ CD11c^int^ Ly6c^hi^ Ly6g^-^ MHC II^hi^), previously described [36,37,38,39]. At day eight, lung populations included in the analysis were general macrophage, neutrophils (SSC^+^ CD11b^+^ CD11c^lo^ Gr-1^hi^), inflammatory monocytes (SSC^-^ CD11b^+^ CD11c^lo^, Gr-1^hi^), CD4+ T cells (CD3ε^+^ CD4^+^ CD8^-^) and CD8+ T cells (CD3ε^+^ CD4^-^ CD8^+^).

Whole lung tissue was homogenized into a single cell suspension by pressing tissue through a strainer into a petri dish, cells were collected into a microtube then centrifuged at 140 x g for five minutes. Red blood cells were removed through hypotonic lysis then cells were washed two times with RPMI media. Cell counts were determined for each sample, then cells were plated at a rate of 2 X 10^6^ cells per well in duplicate. Each well was treated with the following: 1μM of NP peptide, NP_366-374_ (ASNEMNDAM), phorbol myristate acetate (PMA)/Ionomycin as a positive control, or no stimulation (media control) as a negative control for 2 hours at 37°C. Brefeldin (Golgi Plug) was added for 4 hours at 37°C. Cells were pelleted by centrifugation at 140 x g for 5 minutes, resuspended in 100 μL 0.1% BSA PBS wash buffer and stained with rat anti-mouse CD8β Alexa Fluor 488 and rat anti-mouse CD3ε PerCP-Cy5.5. Cells were then fixed, permeabilized and stained using rat anti-mouse IFNγ APC or rat anti mouse TNFα PE. Each sample was analyzed on the FACS Canto to determine the number of CD8β^+^IFNγ^+^ cells or CD8β^+^TNFα^+^ cells activated by the various treatments (PMA/Ionomycin, NP-peptide and no-treatment). FlowJo 7.6 software was used for gating and analysis. Data represent NP-peptide treated wells minus media alone wells.

### Ciliary beat frequency (CBF) determination

Tracheas were removed from mice and placed in serum free M199 medium containing penicillin and streptomycin. Tracheal rings were cut (~1 mm) and placed in six-well tissue culture plates containing serum-free M199 to measure CBF. A baseline CBF measurement was made followed by the addition of procaterol (10 μM). Tracheal rings were incubated for 30 minutes at 37°C in 5% CO_2_ and then allowed to equilibrate at 25°C for 10 minutes, and a final CBF reading was taken. CBF was quantified using the Sisson-Ammons Video Analysis (SAVA) system (46). Whole-field analysis was performed using software that analyzes the entire captured image of all ciliated cells in a given field and the average frequency of cilia (representing thousands of motile points per data point) recorded in at least six separate microscope fields of view. All frequencies are expressed as mean ± SEM from six separate fields.

### IgG and IgG2c ELISA

Blood was collected by heart puncture shortly after euthanization and centrifuged at 180 x g for 15 minutes for serum collection. All sera was stored at -80°C until IAV-specific total IgG and subtype IgG2c antibody ELISA testing could be completed. ELISA plates (Immulon, Alexandria, VA) were coated with 100 μL of influenza virus A/PR/8/34 (200 HAU/mL) diluted in carbonate coating buffer (pH 9.6) and incubated overnight at 4°C. Plates were blocked with 0.1% BSA-PBS blocking buffer at 37°C for one hour. Plates were then washed three times with 0.05% Tween 20-PBS between each incubation period. Diluted sera (1:5–1:50) was added to the plates in duplicate, then incubated for four hours at room temperature. AP-conjugated goat anti-mouse IgG and IgG2c antibodies were added to their respective plates and incubated for eight hours at 4°C. Plates were developed using phosphatase substrate (Sigma Aldrich, St. Louis) for 15 minutes at room temperature. Optical density (OD) of each well was measured using a microtiter plate reader (FluoStar Galaxy) at 405 nm wave length.

### Leptin and IFNα ELISA

Leptin concentration was measured in mouse serum and BAL fluid. A leptin ELISA kit was purchased from R & D Systems (Minneapolis, MN), and the manufacturer’s directions were followed to complete the assay. A mouse IFNα ELISA kit was purchased from Cell Sciences (Canton, MA) to detect IFNα in BAL fluids. The manufacturer’s instructions were followed to complete the assay.

### Statistical Analysis

All statistical analysis was carried out using SPSS Statistics 17.0. A two-way ANOVA (diet by exercise interaction) was used to establish main effects of diet and exercise on immune measures, including antibody titers, BAL cytokines and chemokines, leptin, and cell populations. A mixed ANOVA (diet X exercise, treatment X time) with time as repeated measures (within subjects) variable was used to analyze change in body weight over time. One-way ANOVA was used to determine statistical significance for the mucociliary clearance experiment and microarray analysis. Pearson correlation coefficients were determined to examine correlation between lung viral load and cytokine/chemokines or BAL cell populations. Statistical trends were denoted by *p <* 0.1 and significance levels for all tests were set at *p* < 0.05.

## Results

### Effect of exercise on body fat composition and food intake

**Fig 1:**
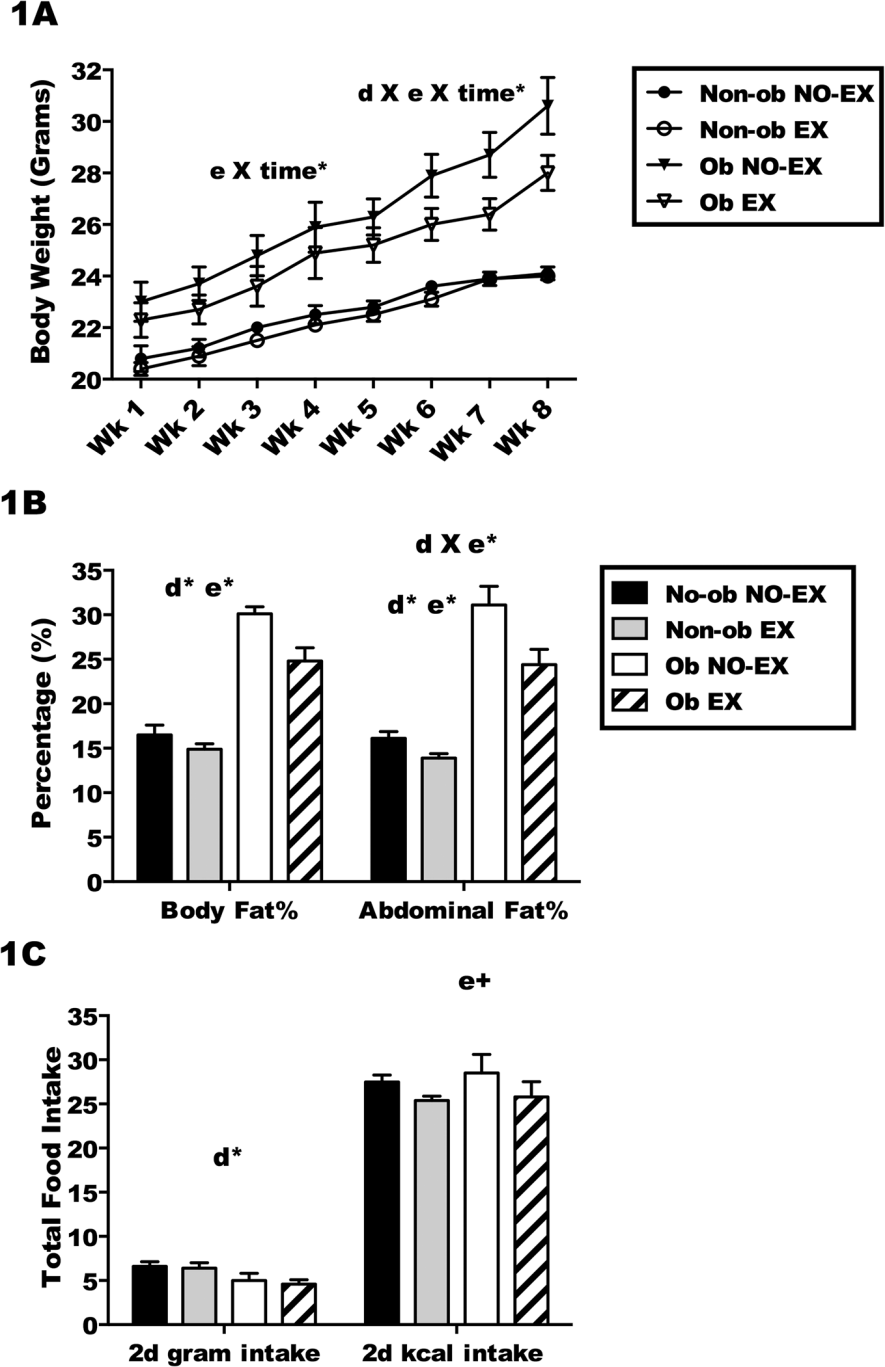
The obese state is established in C57BL/6 mice after eight weeks of high-fat feeding and daily exercise. C57BL/6 mice consumed a high-fat diet (Ob) or low-fat diet (Non-ob) for eight weeks, a subset of mice on each diet exercised (EX) 45 minutes per day, 5 days/week for eight weeks. Sedentary mice on each diet were included as non-exercised controls (NO-EX) 1A.) Body weight (grams) was monitored weekly from week one to week eight for all treatment groups (Non-ob NO-EX, Non-ob EX, Ob NO-EX and Ob EX). 1B). Total body fat and abdominal fat percentage was measured using DXA imaging. 1C) Total food intake (grams) and kilocalorie (kcal) intake was determined over a two day (2d) period prior to infection for all treatment groups. d X e X time* indicates a significant (*p* < 0.05) diet by exercise by time interaction on the change in body weight over time, determined by mixed ANOVA with time as repeated measures (within subjects) variable. e X time* for a significant (*p* < 0.05) effect of exercise over time on change in body weight, by two-way ANOVA with repeated measures within subjects. d X e* for a significant (*p* < 0.05) interaction between diet and exercise. d* for a significant (*p* < 0.05) effect of diet and e* for a significant (*p* < 0.05) effect of exercise (e+; *p* < 0.1). Results are representative of 3 separate experiments. Data are shown as the mean ± SEM. Sample size (n) equals 24–26 mice per group.

Body weight was measured weekly to assess the impact of diet and exercise on weight gain. At 6 weeks of age, one half of the mice began a high fat diet, whereas the other half consumed a matched low fat diet. The mice remained on their respective diets until the conclusion of the study. Half of the mice on each diet treatment exercised regularly (5 days/week, 45 minutes/session, moderate intensity), whereas the other mice did not exercise. The high fat diet model is a standard model to study obesity, and as expected, high fat diet treatment resulted in an obese mouse (average body fat > 23%). Mice receiving the low fat diet were non-obese (average body fat < 16%) (Fig 1). A comparison of the four treatment groups (Non-ob NO-EX), (Non-ob EX), (Ob NO-EX), and (Ob EX), showed that weight gain was greater among the obese mice compared to non-obese mice (Fig 1; d X e X time*, a significant diet by exercise by time interaction; *p* < 0.05). Exercise treatment did impact weight gain, with less weight gain in Ob EX mice compared to Ob NO-EX mice (e X time*; *p* < 0.05). The Non-ob NO-EX mice and Non-ob EX mice gained a similar amount of weight during the training period (Fig 1). Although body weight serves as an estimate of obesity status, given that exercise may result in greater increase in muscle weight as compared to fat, it was important to assess body fat in addition to body weight. After eight weeks of exercise and diet treatment, mice from each treatment group underwent an imaging procedure using a DXA (dual-energy X-ray) scanner to determine total body fat percentage and abdominal fat percentage. As expected, both high-fat fed groups (Ob NO-EX and Ob EX) had significantly higher body fat percentage in comparison to the low-fat fed mice (Non-ob NO-EX and Non-ob EX) (d*; main effect of diet; *p* < 0.05). Exercise treatment resulted in significant reductions in total and abdominal fat percentage (e*; main effect of exercise; *p* < 0.05), with less fat in exercised mice on either diet treatment compared to non-exercised mice (Fig 1). The effect of exercise was slightly different in high-fat diet mice compared to low-fat diet mice such that exercise reduced abdominal fat percentage to a greater extent in high-fat diet mice (d X e*; a significant diet by exercise interaction; *p* < 0.05).

Food intake was compared between treatment groups for two days during the last week of exercise training. When food intake was assessed using total grams (Fig 1), obese mice consumed fewer grams than the non-obese mice (d*; *p* < 0.05), but when kilocalorie intake was assessed, the kilocalorie intake per day was comparable across groups (Fig 1). There was a trend towards reduced calorie consumption in both Non-ob EX and Ob EX mice relative to non-exercised mice (e+; *p* < 0.10).

### Illness severity and lung viral load

**Fig 2:**
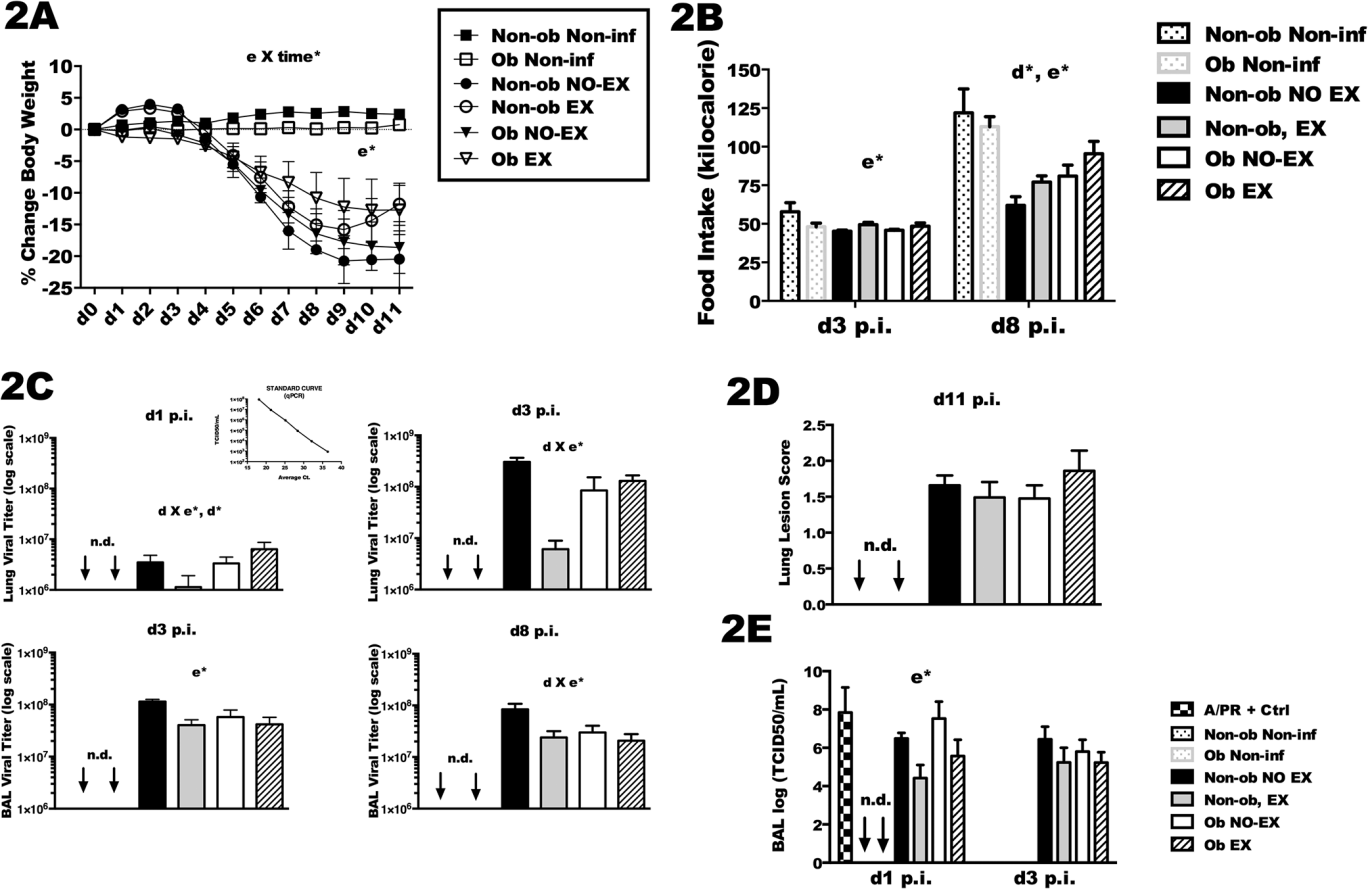
Exercise is associated with improvements in body weight, kilocalorie intake, and reduced lung viral titer after influenza infection. Exercised mice completed an eight week exercise training period followed by i.n infection with A/PR/8/34 (558 EID/mouse) twenty-four hours after the last exercise session. Non-exercised mice (non-obese and obese) were infected at the same time, along with mock-infected (i.n. saline, or non-infected) mice. Subsets from each group were euthanized at day 1, day 3, day 8 and day 11 post-infection (p.i.). 2A) Body weight loss (grams) was monitored through the infection period for all groups. 2B) Food intake (kcal) was monitored as grams of food converted to kilocalorie intake as shown. 2C) Lung tissue was collected at day one (d1) and day three post-infection (d3 p.i.), whereas bronchoalveolar lavage (BAL) was collected at d1, d3 and day 8 p.i. Lung viral quantification was determined by qRT-PCR using primers specific for influenza nucleoprotein (NP) mRNA. Virus of known concentration was used to develop a standard curve (shown in 2C –top inset). Ct values from unknown samples were converted to TCID_50_/mL using the standard curve. 2E) Viral quanitificaton by infectivity (TCID_50_/mL) assay in the indicated lung tissue (whole lung or BAL) was also performed. 2D) Lung lesion scoring were assessed in a blinded fashion from 3–4 lobes per mouse at d11 p.i. Mixed and two-way ANOVAs were used to test for statistical significance of exercise and diet treatments on body weight loss and food intake (2A and 2B). Significant (*p* < 0.05) interactions between exercise and time are indicated by e X time*, with significant (*p* < 0.05) interactions between diet and exercise indicated by d X e*, d* indicates a main effect of diet; *p*< 0.05, and e* for a main effect of exercise treatment; *p* < 0.05. Virus was not-detectable in the non-infected mice as indicated by the acronym ‘n.d.’. For infected mice euthanized at the various time points post-infection sample size (n) is as follows: day one, n = 3–4 per group, day three, n = 8–12, day eight, n = 8–13, and for day eleven, n = 9–12. For non-infected mice sample size (n) equals 1–3 mice per group. Data are represented as the mean ± SEM from two separate experiments.

Illness severity was assessed using body weight loss and total kcal intake. All IAV infected mice lost a significant amount of weight in comparison to mock-infected mice during the observation period of the study (Fig 2). Body weight loss was expressed as percent change in body weight compared to pre-infection body weight. Exercised mice (Non-ob EX and Ob EX) lost less weight than non-exercised mice (Non-ob NO-EX and Ob NO-EX) (e*; *p* < 0.05) (Fig 2). Also, a significant interaction between exercise and time was found revealing that the weight loss over the course of infection was reduced in exercised mice (e X time*; *p* < 0.05). Food intake declined during infection as expected, and the total amount of food consumed by Non-ob EX and Ob Ex was significantly greater than non-exercised (Non-ob and Ob) in two separate experiments (Fig 2). In the experiment in which mice were euthanized at day 3 post-infection (d3 p.i.), total kilocalorie intake was increased in exercised mice (e*; *p* < 0.05). Similarly, in mice euthanized at day eight post-infection (d8 p.i.) total kilocalorie intake was greater in exercise-treated mice on either diet (e*; *p* < 0.05). When food intake was summed over eight days, it was apparent that obese mice consumed more food than non-obese mice (d*; *p* < 0.05), although food consumption in all treatment groups was reduced relative to non-infected control mice. When food intake was calculated as kilocalorie per gram of body weight instead of total kcal, exercised mice also tended to have a greater caloric intake relative to non-exercised mice during the weight loss phase of the infection (days 5–9, S1 Fig). However, kcal consumption by obese mice was half that of non-obese mice when totaled during the weight loss phase (days 5–9). Caloric intake continued to decline in non-exercised obese mice at day 8 and 9, but had begun to increase in all other groups (S1 Fig).

Lung tissues collected at day one post-infection (d1 p.i.) and d3 p.i. were used to determine lung viral load (Fig 2), whereas viral load at d3 and d8 was evaluated in the bronchoalveolar lavage (BAL) fluid. BAL viral titer and lung viral titer were highly correlated with one another at day 3 (Pearson correlation coefficient = 0.806, *p* < 0.001) (S2 Fig). At d1, obese mice had higher viral loads than non-obese mice (d*; *p* < 0.05). However, the effect of exercise was different in obese and non-obese mice with reduced viral load in Non-ob EX mice, but increased viral load in Ob EX mice (d X e*; *p* < 0.05). Exercise treatment reduced viral load at d3 p.i. and d8 p.i., an effect of exercise only observed in the non-obese mice (d X e*; *p* < 0.05). In a subset of mice euthanized at day 1 and day 3 post-infection, lung viral load was also assessed using a viral infectivity assay. The results (TCID_50_/mL) are similar to viral load quantified by qRT-PCR, except that a trend towards reduced virus in exercised obese mice was observed on d1 p.i. (Fig 2). Finally, the extent of lung lesions was assessed in mice euthanized at d11 p.i., but at this time point no differences were found among the treatment groups even though these were the same mice in which reduced illness severity was observed (Fig 2).

### Ciliary beating is increased by exercise in obese mice

**Fig 3:**
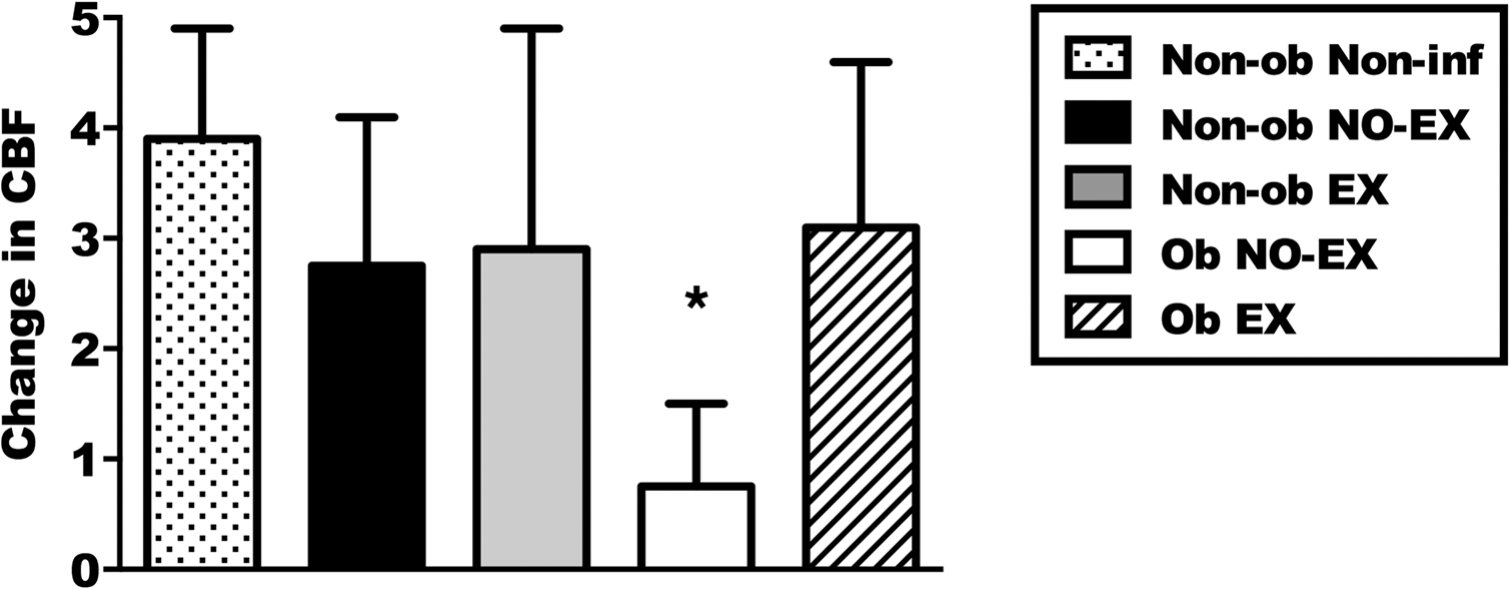
Exercise maintains ciliary beat frequency (CBF) in the tracheas of influenza-infected obese mice. Tracheas were quickly excised from euthanized mice then treated with the beta-agonist, procaterol, to stimulate cilia movement on airway epithelial cells. A significant (*; *p* < 0.05) effect of exercise in obese mice was determined by a one-way ANOVA and Tukey’s post-test. Tracheas from ten mice were included from each of the infected groups euthanized at day three p.i. Three tracheas from non-infected mice were included as positive CBF control. Data are shown as the mean ± SEM. Results represent two separate experiments.

Tracheas were collected at d3 p.i. and d8 p.i. and stimulated with the beta-agonist procaterol to stimulate ciliary beat frequency (CBF) in epithelial cells (S1 Video). Changes in CBF equate to changes in mucociliary clearance. CBF was quantified per minute and represented in Fig 3. In general, IAV infection results in a reduction of CBF, and by d8 p.i., all infected mice had significant epithelial damage such that CBF could not be determined. However, in comparison to infected mice from the different treatment groups at d3 p.i., CBF was increased with exercise treatment in obese mice only (* significant effect of exercise; *p* < 0.05). These results show that the obese condition was associated with a decreased CBF, but exercise restored CBF to a level observed in non-obese mice.

### Lung cell populations in response to IAV infection

**Fig 4:**
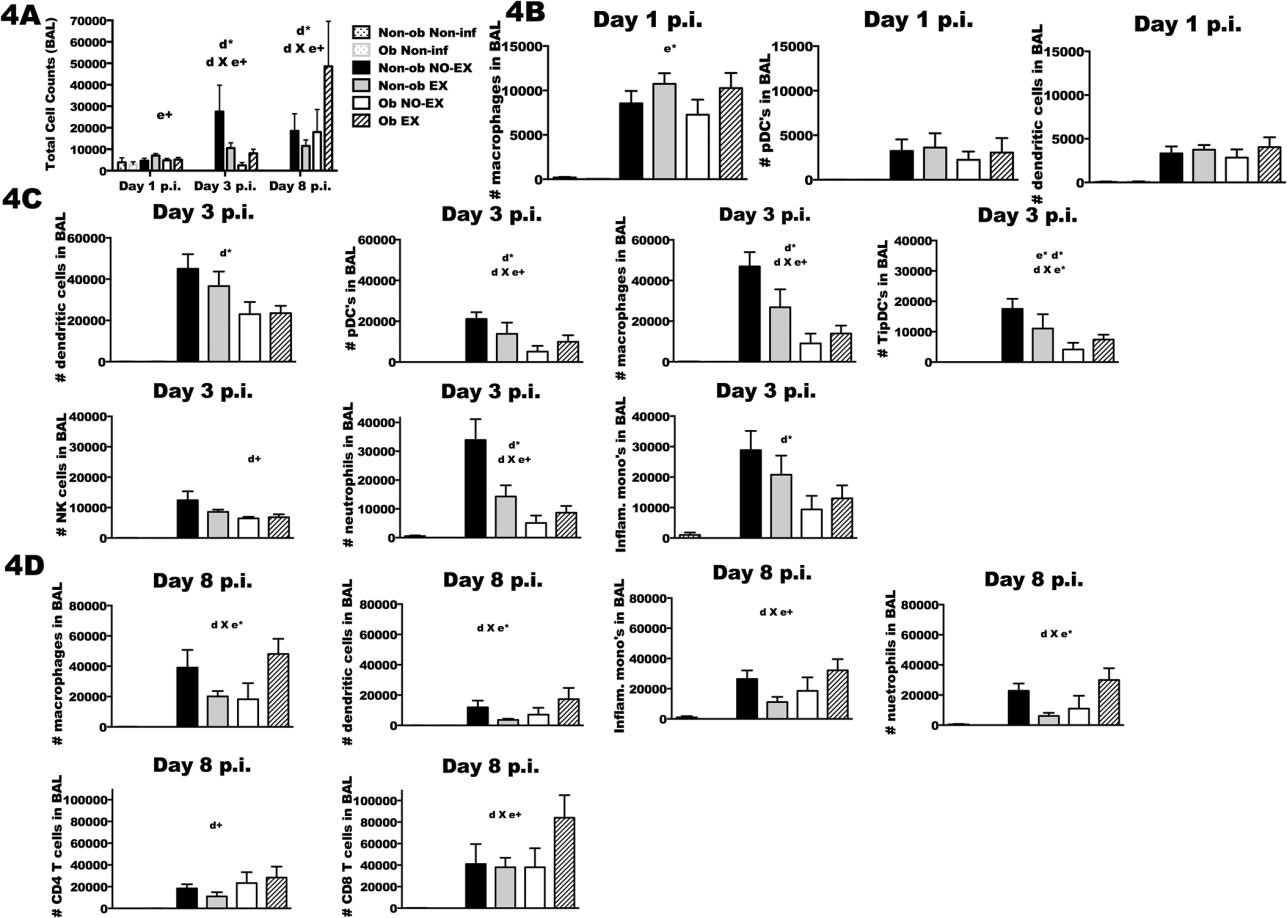
Immune cell infiltration is altered by obesity status and exercise treatment. Mice from each treatment group were infected with A/PR/8/34 then euthanized at day one, day three and day eight post-infection (p.i.). Non-obese and obese non-infected mice were included as controls. Total cell infiltration was quantified and specific immune cell populations were characterized in bronchoalveolar lavage (BAL) fluid at each indicated time point. 4A) Total BAL cell numbers were quantified by flow cytometry using FSC and SSC. 4B) At d1 p.i., number of macrophages, plasmacytoid dendritic cells (pDC) and dendritic cells were determined by flow cytometry, along with, 4C) dendritic cells, pDC, macrophages, TNFα/iNOS producing dendritic cells (TipDC), natural killer (NK) cells, neutrophils and inflammatory monocytes at d3 p.i. 4D) At d8 p.i., macrophage, dendritic cells, inflammatory monocytes, neutrophils, CD4+ T cells and CD8+ T cells were quantified similarly using flow cytometry. The acronym ‘n.d.’ indicates that the cell population was not-detectable in the non-infected group. Based on the results of a two-way ANOVA, a significant (*p* < 0.05) interaction between diet and exercise treatments is represented by d X e* (d X e+; *p* < 0.1), d* indicates a significant (*p* < 0.05) effect of diet (d+; *p* < 0.10), and e* indicates a significant (*p* < 0.05) effect of exercise (e+; *p* < 0.10). For infected mice euthanized at the various time points post-infection sample size (n) is as follows: day one, n = 3–4 per group, day three, n = 8–12, day eight, n = 8–13, and for day eleven, n = 9–12. Sample size (n) equals 1–3 mice for the non-obese and obese non-infected groups. Data shown as mean ± SEM. Results are representative of two separate experiments.

**Fig 5:**
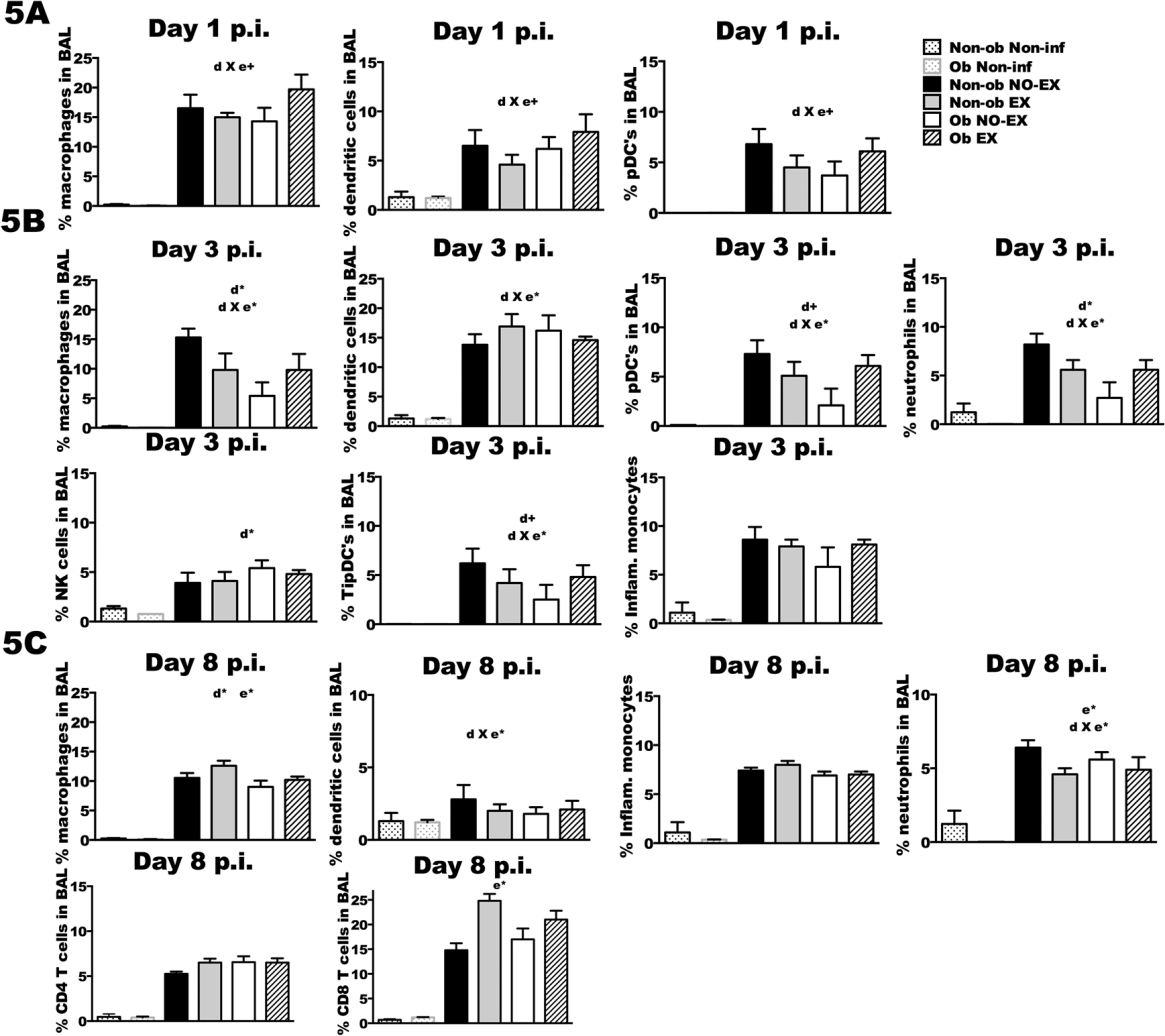
Lung cell composition in response to influenza infection is altered by exercise treatment in obese and non-obese mice. Subsets of mice from each treatment group were infected with A/PR/8/34 then euthanized at the indicated time points post-infection. Total cell infiltration was quantified and percentages of the specific immune cell populations were characterized in bronchoalveolar lavage (BAL) fluid using flow cytometry. The percentage of each cell population is expressed as percent of gated parent population per total BAL cells. 5A) Percentage of macrophages, dendritic cells, and plasmacytoid dendritic cells (pDC’s) were detected at d1 p.i., 5B) macrophages, dendritic cells, pDC’s, neutrophils, NK cells, TNFα/iNOS producing dendritic cells (TipDC’s) and inflammatory monocytes were similarly detected at d3 p.i. 5C) At d8 p.i., the percentage of macrophages, dendritic cells, inflammatory monocytes, neutrophils, CD4+ T cells and CD8+ T cells were also determined. The acronym ‘n.d.’ indicates that the cell population was not-detectable in the non-infected group. Based on the results of a two-way ANOVA, a significant (*p* < 0.05) interaction between diet and exercise (d X e*) was determined (d X e+; *p* < 0.1); d* indicates a significant main effect of diet at *p* < 0.05, (d+; *p* < 0.10); e* indicates a significant main effect of exercise at *p* < 0.05 (e+; *p* < 0.10). Sample size (n) is 5–13 per infected group; n = 1–3 for the non-infected groups. Data shown as mean ± SEM. Results are representative of two separate experiments.

BAL fluid was collected for total cell counts (Fig 4), the total number of specific cell populations (Fig 4), and the percentages of infiltrating immune cells (Fig 5). With respect to number of cells, exercise resulted in a modest increase in cell number in both obese and non-obese mice at d1 p.i. (e+; *p* < 0.1). The effect of exercise at later time points varied by obesity status (d*; *p* < 0.05); Non-ob EX mice had significantly fewer cells recruited to the lungs relative to Non-ob NO-EX, whereas Ob EX mice had a greater number of cells recruited than Ob NO-EX mice (Fig 4) (d3 p.i.; d X e+; *p* < 0.1 and d8 p.i.; d X e*; *p* < 0.05). Cell recruitment was highly correlated with lung viral load in non-obese mice (d3 p.i., Pearson correlation coefficient = 0.420, *p* < 0.001; at d8 p.i., Pearson correlation coefficient = 0.123, *p* = 0.01) and therefore, in non-obese mice the reduction in cell number resulting from exercise treatment may reflect decreased viral load (S3 Fig). However, in obese mice, the typical relationship between lung viral load and cell recruitment was not present, suggesting dysregulation in the recruitment of cells in proportion to viral load. At d3 p.i., obese mice had fewer macrophages, conventional dendritic cells (cDC), plasmacytoid dendritic cells (pDC), TNFα/iNOS producing dendritic cells (TipDC), natural killer cells (NK), inflammatory monocytes, and neutrophils compared to non-obese mice (Fig 4) (d*; *p* < 0.05 and d+; *p* < 0.1). Exercise treatment of obese mice partially restored cell recruitment for all cell types except cDC and NK cells at day 3 (e*; *p* < 0.05, d X e*; *p* < 0.05 and d X e+ *p* < 0.1). By d8 p.i., exercise completely restored the number of macrophages, cDC, inflammatory monocytes, neutrophils, and actually increased the number of CD8+ T cells relative to non-obese mice (d X e+; *p* < 0.1) (Fig 4).

Exercise training altered the composition of infiltrating cells, and again this response varied by obesity status. Exercise reduced the percentage of macrophages, DC, and pDC at d1 p.i. in non-obese mice (Fig 5). Similarly in non-obese mice, the percentage of macrophage, pDC, neutrophils, and TipDC in the BAL was decreased in exercise-treated mice at d3 p.i., only an increase in the number of cDC was observed (Fig 5). These changes may reflect reduced viral titer in Non-ob EX mice. At d8 p.i., exercise increased the percentage of CD8+ T cells and macrophages, but reduced neutrophils in non-obese (Fig 5). The exercise-related increase in CD8+ T cells likely reflects improved viral clearance as a greater percentage of CD8+ T cells in the BAL was significantly correlated with decreased viral load (S4 Fig). The greater percentage of macrophages was associated with reduced viral load as well, suggestive of the possibility that macrophages contributed a repair role rather than an inflammatory role at this stage of infection (S5 Fig). Exercise treatment in obese mice had an opposite effect, particularly at d1 and d3 p.i. (Fig 5). Exercise in obese mice resulted in a greater percentage of macrophages and pDC at d1 and d3 p.i., and TipDC and neutrophils at d3 post-infection. In Ob EX mice, a greater percentage of these same BAL cell populations were associated with higher viral load, this same relationship with viral load was not found in Ob NO-EX mice suggesting that exercise “restored” the normal immune response to IAV infection. At d8 p.i., although total number of cells differed between Ob EX and Ob NO-EX, the composition of the BAL populations was similar, with the only difference being a slight increase in the percentage of CD8+ T cells in the exercised mice (Fig 5).

### BAL chemokine and cytokine concentrations vary by exercise treatment and obesity status

**Fig 6:**
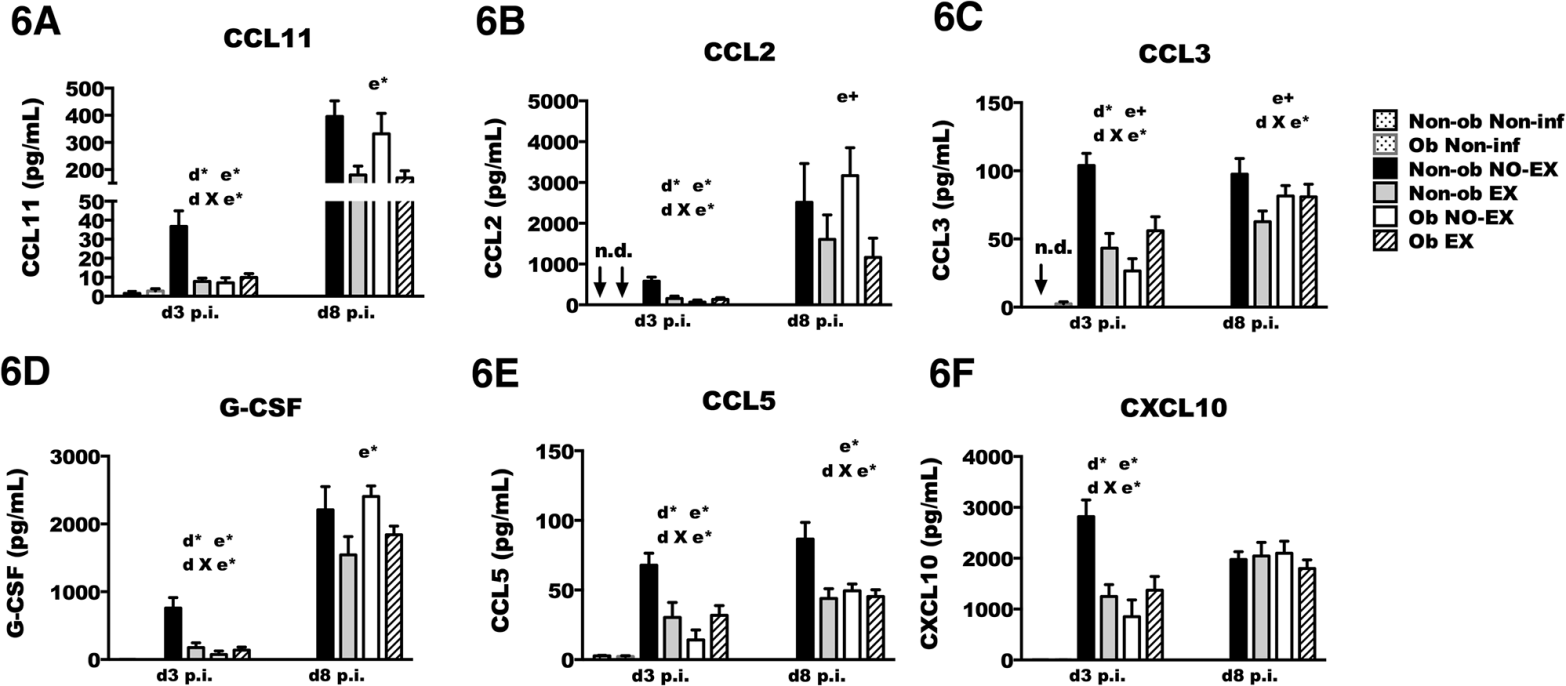
Chemokine concentration in BAL is altered by exercise and obesity. Mice from each treatment group were infected with A/PR/8/34 then euthanized at day three (d3) or day eight (d8) post-infection (p.i.). BAL concentration (pg/mL) of various chemokines was detected by a multiplex assay on the Luminex platform. The acronym ‘n.d.’ denotes that the protein was not-detectable in the non-infected group. A significant (*p* < 0.05) interaction between diet and exercise was determined by two-way ANOVA; indicated by d X e* in the figure. A main effect of exercise is indicated by e* (*p* < 0.05) (e+; *p* < 0.1). A main effect of diet is signified by d* (*p* < 0.05) (d+; *p* < 0.1). Sample size (n) equals 8–13 mice per infected group; non-infected mice were included as controls (n = 1–3). Data shown as mean ± SEM. Results are representative of two separate experiments.

The alterations of cell recruitment to the lungs could be influenced by chemokine concentration. In this study, chemokine production in obese mice was impaired relative to non-obese mice, primarily at the early stages of IAV infection (Fig 6, S1 Table and S2 Table). The chemokine response pattern mimicked the findings with respect to total cell number recruited to the BAL, such that exercise treatment reduced chemokine concentration in non-obese mice, but generally increased chemokine concentration in obese mice (d X e*; *p* < 0.05). Similar to the findings with respect to BAL cell numbers, in non-obese mice the chemokine concentration was correlated with viral load (S6 Fig), which may explain the reduction in chemokine levels. In obese mice, it appears that there was a delay in chemokine production rather than an absolute impairment given that by d8 p.i., chemokine levels were comparable in non-obese mice and obese mice. Exercise treatment improved the obesity-associated impairment of chemokine production at d3 p.i. The chemokine concentration for CCL2, CCL3, CCL4, CCL5, CCL11, CCL12, CCL19, CCL20, CXCL1, CXCL9, and CXCL10, was ~ 2–3 fold greater in Ob EX mice compared to Ob NO-EX (Fig 6 and S1 Table). At d8 p.i., there was no longer an obesity-associated decrease in chemokine concentration. Instead, CCL2 (MCP-1), CCL11 (eotaxin), G-CSF, and CCL4 (MIP-1β) were decreased by exercise regardless of obesity status. The chemokines CCL3 (MIP-1α) and CCL5 (RANTES) were decreased only in exercise-treated non-obese mice (Fig 6). These findings may suggest that exercise treatment limited further inflammatory cell influx at this stage of infection, potentially a consequence of enhanced viral clearance due to the greater concentration of CD8+ T cells in the BAL.

**Fig 7:**
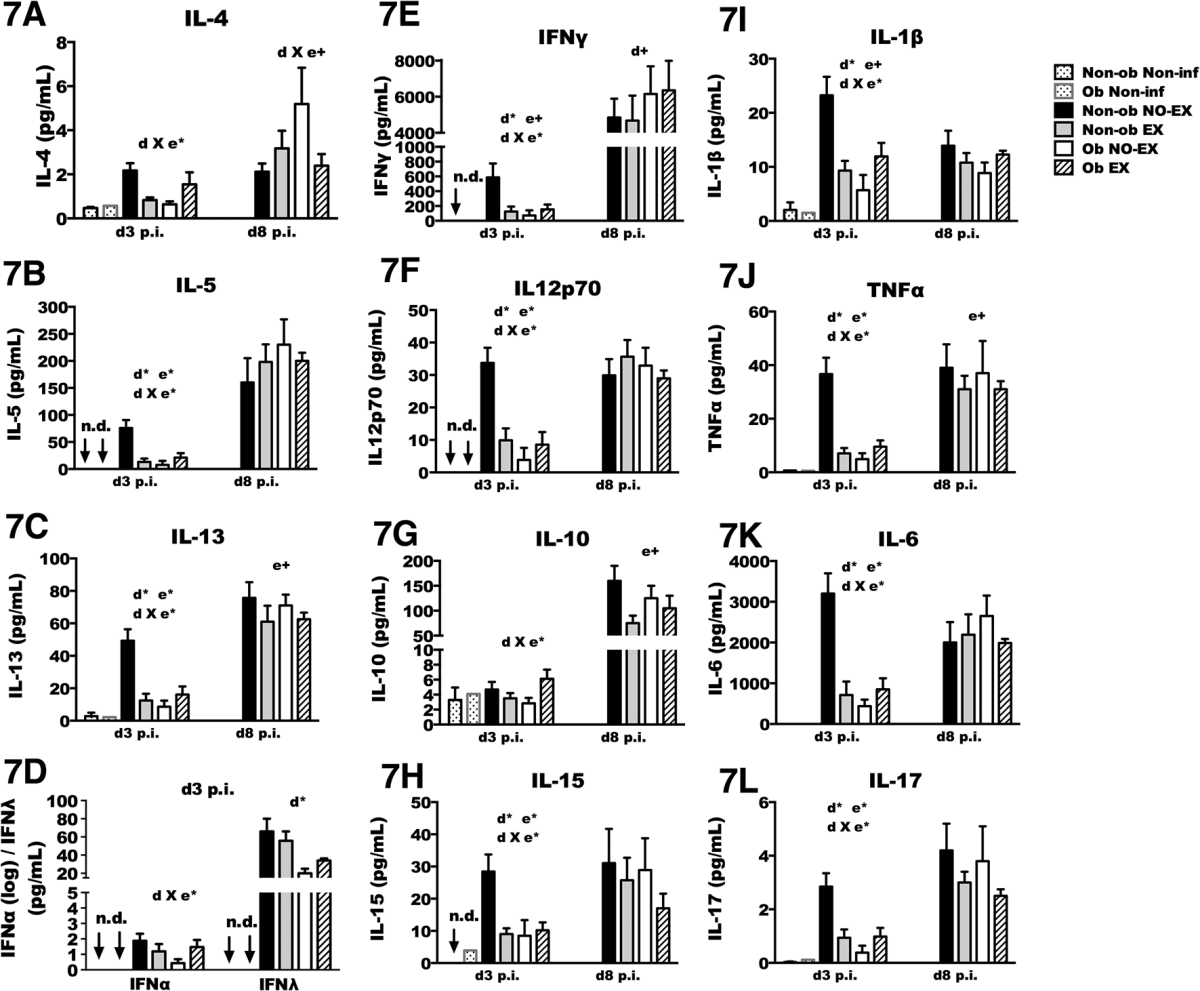
Cytokine concentration in BAL is altered by exercise and obesity. Mice from each treatment group were infected with A/PR/8/34, and then euthanized at the indicated time points. BAL cytokine concentration (pg/mL) was assessed by a multiplex assay on the Luminex platform. The acronym ‘n.d.’ denotes that the protein was not-detectable in the non-infected group. Two-way ANOVA was used to test for statistical significance; d X e* indicates a significant (*p* < 0.05) interaction between diet and exercise (d X e+; *p* < 0.1). A main effect of exercise is indicated by e* (*p* < 0.05) (e+; *p* < 0.1). A main effect of diet is signified by d* (*p* < 0.05) (d+; *p* < 0.1). Sample size (n) equals 8–13 mice per infected group; for non-infected groups n = 1–3. Data shown as mean ± SEM. Results are representative of two separate experiments.

**Fig 8:**
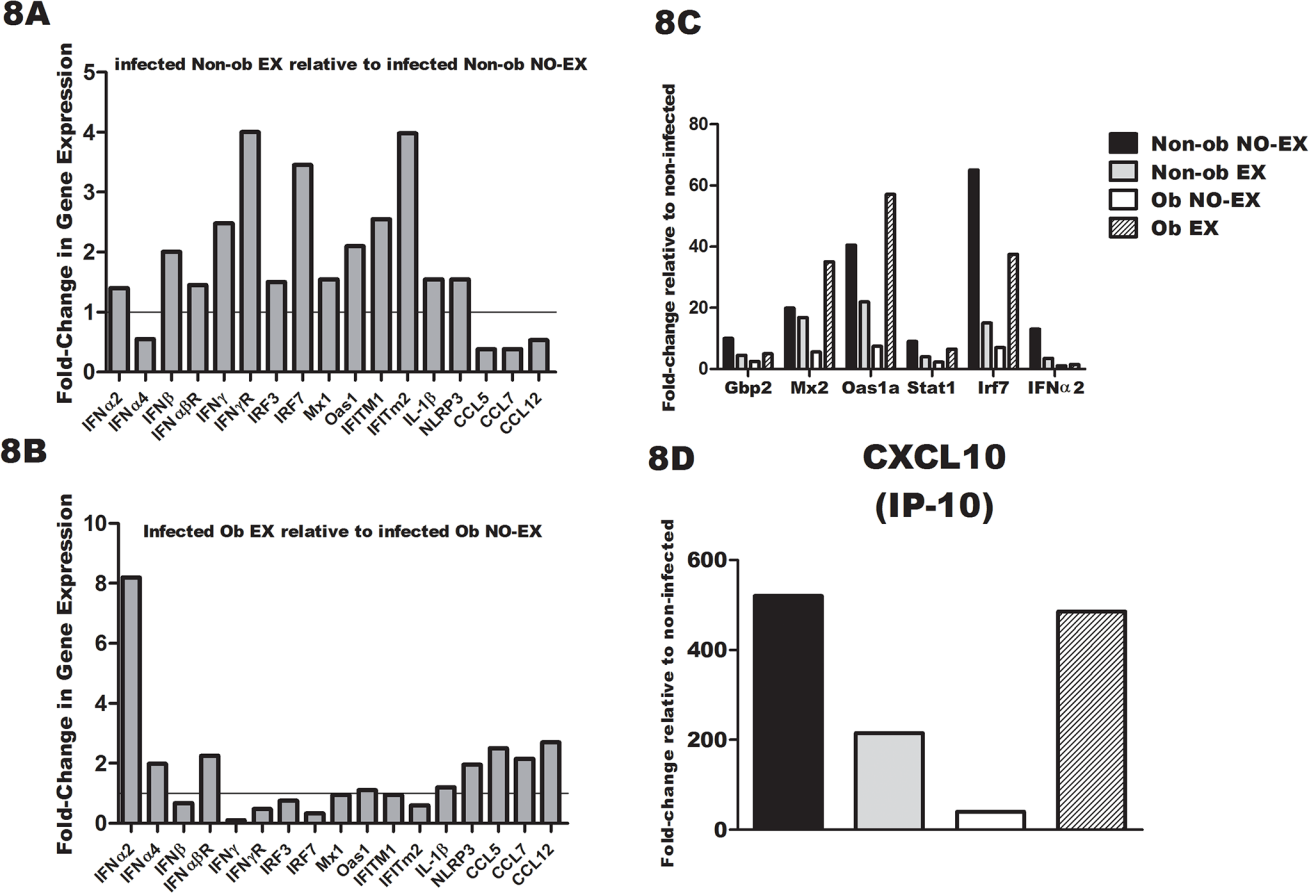
Exercise treatment restores IFN-α and IFN-stimulated gene expression in obese mice. 8A) Interferon-stimulated genes or inflammasome-related genes measured by PCR microarray in mRNA isolated from the lungs of non-obese mice at day one p.i. The fold-change shown reflects non-obese exercise mice relative to non-obese non-exercise mice. 8B Results in figure represent fold change in gene expression of obese exercise mice relative to obese non-exercise mice on day one p.i.8C and 8D) Interferon stimulated genes measured by PCR microarray in mRNA isolated from the lungs of obese-mice and non-obese mice that did or did not exercise on day three p.i. The fold-change shown is relative to non-infected mice for each treatment group. Sample size was 4–6 per group.

Cytokine concentration was also measured in BAL fluid (Fig 7). Early during infection (i.e., d3 p.i.), obesity was again associated with reduced production of cytokines IL-13, IL-5, IL-12p70, IFNγ, TNFα, IL6, and IL-1β, as well as IL-28b (IFNλ), IL-17, and IL-15. IL-4 and IL-10 were not reduced in obese mice. Similar to findings of cell recruitment and chemokine production, again non-obese and obese mice responded differently to exercise as Non-ob EX mice had reduced cytokine concentration whereas Ob EX mice had increased levels of IL-1β, IFNγ, IFNα, IFNλ, IL-6, IL-5, IL-10, IL-12p70, IL-13, IL-17 and TNFα relative to Ob NO-EX mice. Exercise treatment reduced cytokines by 2–3 fold or more in non-obese mice, and increased cytokines by ~ 2 fold or more in obese mice. Although the reduced viral load in Non-ob EX mice may have resulted in lower cytokine activation, in obese mice the viral load was nearly equivalent between Ob EX and Ob NO-EX mice. Therefore, viral load alone cannot account for the > 2 fold increase in cytokine concentration in Ob EX mice. In unpublished findings, we have observed that IFNα during the first four days of infection was necessary for optimal cytokine production, and therefore the role of IFNα was examined in subsequent experiments (Fig 8).

At d8 p.i., the obesity-related impairment of cytokine production was no longer present, suggesting that obesity may contribute to a delay in cytokines, rather than an absolute deficiency. In fact, IFNγ concentration tended to be higher in obese mice in comparison to non-obese mice at this time point (Fig 7). Exercise treatment was associated with decreased IL-10 and TNFα in obese and non-obese mice, but did not have a significant impact on the concentration of other cytokines (Fig 7).

### Lung IFNγ- and TNFα-producing CD8+ T cells specific for influenza viral NP-peptide are altered by obesity and exercise status

**Fig 9:**
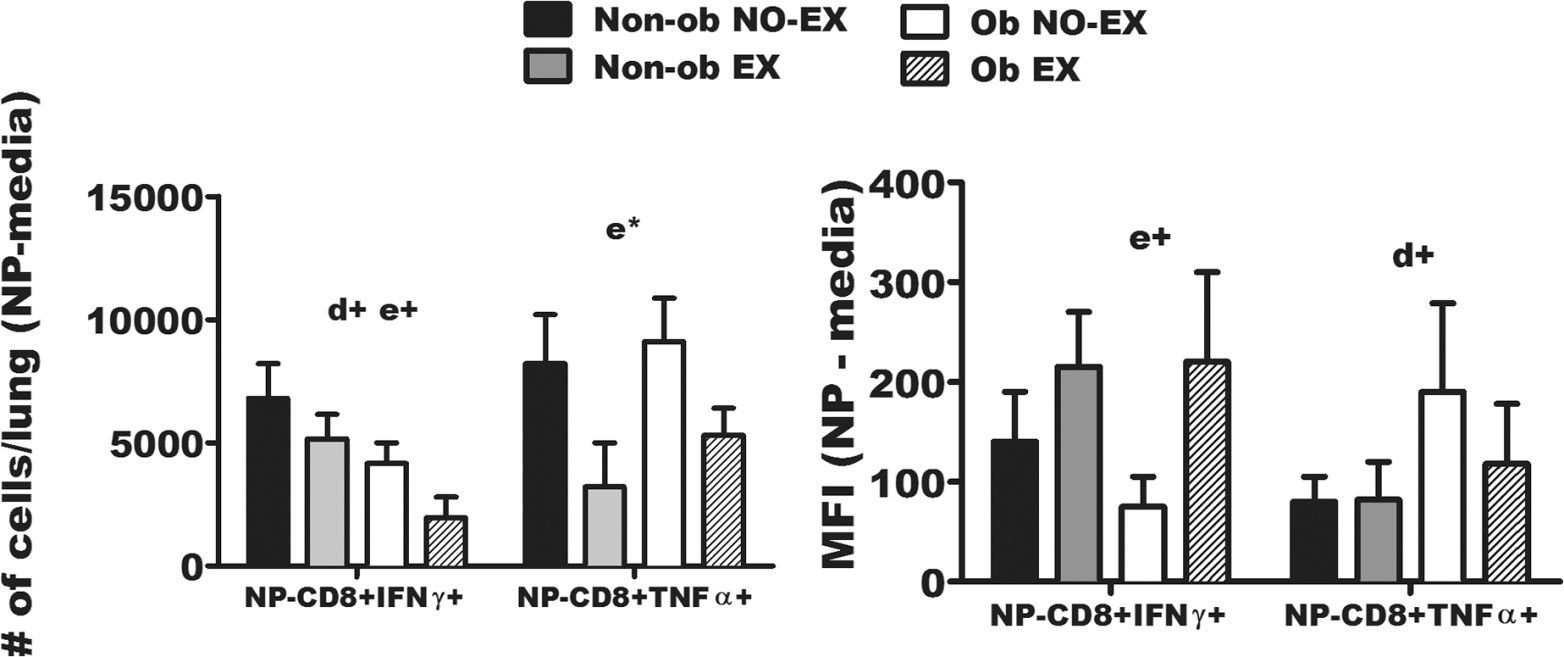
Lung IFNγ- and TNFα-producing CD8+ T cells specific for NP-peptide are altered by obesity and exercise. Lung tissue was collected from influenza-infected mice at d8 p.i. One lung lobe from each mouse was homogenized to determine the magnitude of influenza virus nucleoprotein (NP)-specific CD8+ T cell responses in each group, as determined by intracellular IFNγ or TNFα production detected by flow cytometry. Lung cells were cultured with influenza NP peptide or media alone. The results reflect the NP-specific response minus media alone control wells. The total NP-specific CD8^+^ T cells or media-alone treated cells were normalized to 5 X 10^5^ lung cells. The total number of NP-specific CD8+ IFNγ+ cells and NP-specific CD8+ TNFα+ cells are shown (left-side of figure)), and IFNγ and TNFα MFI (right-side of figure) was determined, respectively. Two-way ANOVA was used to test for statistical significance. A main effect of exercise is indicated by e* (*p* < 0.05), and e+; *p* < 0.1. A trend for a main effect of diet is signified by d+; *p* < 0.1. Sample size (n) equals 8–13 mice per group. Data are shown as mean ± SEM. Results are representative of one experiment.

CD8+ IFNγ-producing T cells and CD8+ TNFα-producing T cells were detected in whole lungs on d8 p.i. in IAV infected mice by *in vitro* stimulation with influenza virus nucleoprotein (NP) peptide or media alone (Fig 9). The data shown represents the response in influenza viral NP peptide stimulated wells minus media alone treated wells. Although exercise treatment tended to decrease the total number of NP-specific CD8+ IFNγ+ T cells (e+; *p* < 0.1) (Fig 9, left), the mean fluorescence intensity (MFI) of IFNγ in NP-specific CD8+ T cells was increased by exercise (e+; *p* < 0.1) (Fig 9, right), suggesting that exercise treatment increased the amount of IFNγ produced on a per cell basis. This finding was consistent in both obese and non-obese mice. However, the total amount of IFNγ in BAL fluid was previously shown to be increased in obese mice (Fig 7), and therefore, total IFNγ in the BAL of obese mice likely reflects production by cells other than antigen-specific CD8+ T cells.

The total number of CD8+ TNFα+ cells responding to influenza viral NP peptide was significantly decreased in exercise treated mice (both obese and non-obese) (Fig 9, left). Exercise treatment did not significantly affect the amount of TNFα produced on a per-cell basis as measured by MFI (Fig 9, right).0020Although the significance of the exercise-related difference in the shift toward greater NP-stimulated CD8+ IFNγ+ T cells and fewer CD8+ TNFα+ T cells remains to be established, others have demonstrated that CD8+ TNFα+ T cells contribute to greater influenza-associated immunopathology [40]. Therefore, this response may serve as another means by which exercise contributes to reducing inflammation-associated tissue damage.

### Anti-influenza virus-specific antibody in response to IAV infection and exercise in obese mice

**Fig 10:**
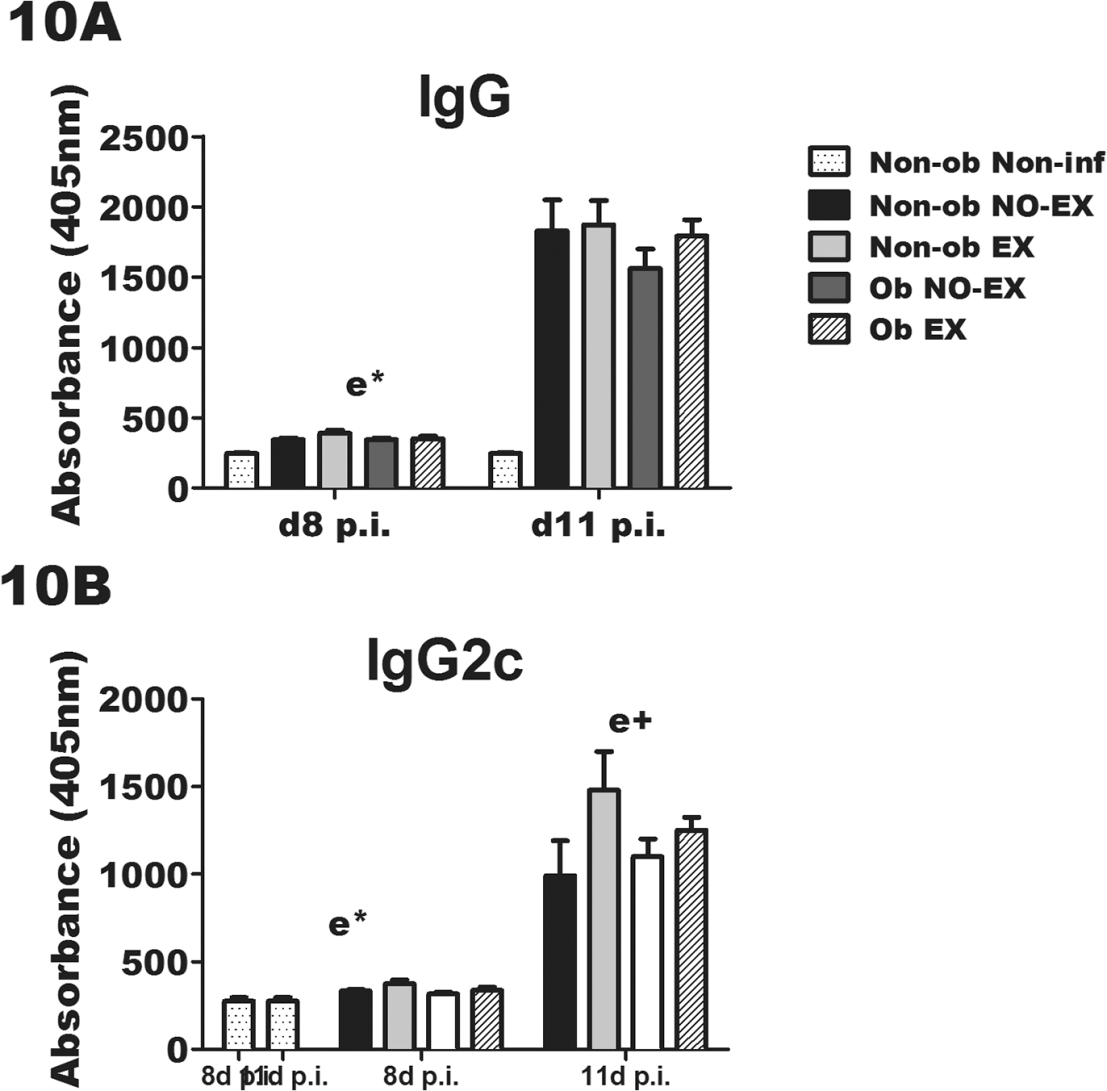
Exercise treatment increases serum anti-influenza antibody in both obese and non-obese mice. Serum anti-influenza 9A) IgG andIgG2c was determined by antibody ELISA in mice euthanized on d8 p.i. (10A and 10B –left side) and d11 p.i. (10-A and 10B –right side). Statistical significance was determined by two-way ANOVA. A significant (*p* < 0.05) main effect of exercise is indicated by e* (e+; *p* < 0.10). Sample size equals 8–12 mice per group; non-infected mice are included as controls and n = 1–3 mice for these groups. Data are shown as mean ± SEM and representative of two separate experiments.

The concentration of anti-influenza virus-specific IgG was determined in mice euthanized on d8 p.i. and d11 p.i. Serum IgG was higher in all infected groups in comparison to the non-infected mice as expected (Fig 10). Exercised mice had greater serum levels of anti-influenza virus-specific IgG at d8 p.i. (e*; *p* < 0.05), with a similar pattern at d11 p.i. (Fig 10). Exercise increased anti-influenza virus-specific IgG2c in both obese and non-obese mice at d8 p.i. (e*; *p* < 0.05), with a trend towards elevated levels on d11 p.i. (e+, *p* < 0.10) (Fig 10). The immunomodulatory effects of exercise tended to differ between obese and non-obese early during infection, and not surprisingly the subsequent antibody response followed.

### Interferon-stimulated and inflammasome-related gene expression varies by obesity level and exercise treatment

Diet-induced obesity impairs immune anti-viral response by delaying the Type I interferon (IFN) response [13,14,15,17,41]. In previous published work, exercise minimized viral load as early as 12 hours post IAV infection [31]. Our unpublished findings, indicate that the Type I interferons, specifically IFNα, may be in part responsible for the exercise-induced improvements in the immune response to influenza viral infection. We therefore examined gene expression for several important interferon-related genes (IFNα2, IFNα4, IFNβ, IFNαβR, IFNγ, IFNγR, Irf3, Irf7, Mx1, Oas1, IFITM1, IFITM2), along with inflammasome-related genes IL-1β and NLRP3, and chemokines CCL5, CCL7, and CCL12 at d1 post-infection. In preliminary experiments, the expression of these specific genes was increased between 24–72 hours post-infection. A different effect of exercise was observed in non-obese and obese mice. In non-obese mice euthanized at day one p.i. (Fig 8), exercise treatment resulted in a modest increase in the expression of IFN-related genes, but a *decrease* in chemokine genes. In contrast, in obese mice the exercise treatment increased IFNα expression by ~ 8 fold at day one p.i., with a modest *increase* in chemokine expression (~ 2 fold) (Fig 8). The results in Fig 8 represent Type I-IFN gene expression relative to non-infected mice at 72 hours post-infection. The effect of exercise differed in non-obese and obese mice, and by this time point, non-obese mice tended to have reduced levels of Type I IFN-related gene expression, but obese exercised mice showed greater levels of gene expression. It is possible that the kinetics of IFN-related gene expression was changed by exercise, but this may vary by obesity state. In non-obese mice, exercise tended to increase IFN-associated gene expression at d1 p.i., but peak expression may have already occurred by d3 post-infection. In contrast, IFN-inducible gene expression appeared to be delayed by obesity, and although exercise countered this effect, it was not apparent until day 3 after infection. In obese mice, the exercise-induced increase in IFNα2 at d1 p.i. may contribute to the increase in IFN-inducible gene expression (Oas1, Mx2, STAT1 and Irf7) (Fig 8). Of all genes evaluated, CXCL10 expression was induced to the greatest extent in infected mice at d3 p.i. (> 500 fold increase) and again, the pattern of decreased expression in Non-ob EX mice, but increased expression in Ob EX mice was observed (Fig 8). The pattern of chemokine expression, as determined in the PCR array, mimicked that of the protein response measure previously in the BAL fluid at early time points (Fig 6 and S1 Table). Corresponding protein data for the following genes is shown in the text as follows: CCL5 and CXCL10 are shown in Fig 6, IFNα and IL-1β are shown in Fig 7, and CCL12 is shown in S1 Table.

## Discussion

Evidence from multiple studies indicates that obesity may increase both susceptibility to and severity of influenza viral infection [7,8,13,14,15,27,42]. Yet, permanent weight loss remains a challenge for a significant proportion of the population. If, in spite of the obese condition, a health practice such as regular moderate exercise can improve host defense against respiratory infection, then a significant percentage of the population may benefit from this practice even under circumstances in which weight loss does not occur. The results of these experiments demonstrate for the first time that exercise reversed several of the impairments of host immunity that have previously been documented to occur in the obese condition. A novel finding in this study was that the exercise-induced immune alterations differed between obese and non-obese mice. Exercise generally increased immune activation (BAL cytokine, chemokine, and cell infiltration) in obese mice early during infection, but had the opposite effect of reduced cell infiltration, and diminished cytokine/chemokine response in non-obese mice at this same time point. There are several possible reasons for these opposing findings including an exercise-associated reduction in viral load in non-obese mice only, as well as a different pattern of interferon-associated gene expression by 24 hours post-infection. Overall the findings indicate that exercise is of benefit in both non-obese and obese mice, albeit through different mechanisms.

Obesity impacts several immune responses to influenza viral infection. In general, during the first several days of infection, IFNα, IFNβ, pro-inflammatory cytokines, and chemokines are reduced in the lungs of obese mice [13,14,15,16,17]. The results of this study are similar to previous observations showing that BAL cytokines and chemokines (IL-1β, TNFα, IL-6, IFNγ, IL-5, GM-CSF, G-CSF, and CXCL10) were decreased in obese mice at d3 p.i., and we have added to the previous research by showing that IFNλ (IL-28b), IL-13, and IL-17, as well as the chemokines CCL2, CCL3, CCL5, CCL11, and CXCL9 were also reduced in obese mice. IFNλ is a Type III IFN that may be the predominant type produced in the respiratory tract during influenza viral infection [43], and therefore this finding adds to the accumulating evidence that obesity significantly impairs the early IFN response in the lung. The reduction in Type I IFN may be one mechanism that contributes to the impaired chemokine response in obese mice, given that IFNα priming of lung epithelial cells impacts subsequent expression of CCL2, CCL5, and CXCL10 [44]. In contrast to the obesity-associated decrease present during the first several days of infection, by d8 p.i., obesity was no longer associated with reduced BAL cytokines and chemokines suggesting that obesity delayed the response, rather than completely inhibiting their production.

Similar to other studies [15,17], we observed that at d3 p.i., obese mice had decreased cell infiltration with fewer total BAL cell, and reduced numbers of macrophages, pDC, neutrophils, NK cells, and inflammatory monocytes in the BAL. It is likely that delayed chemokine expression resulted in delayed cellular recruitment to the lungs. In addition to reductions in these cell populations, obese mice had fewer TipDC, a cell that has been shown to be essential in stimulating CD8+ T cell proliferation necessary for appropriate viral clearance [36]. It is possible that the reduced number of TipDC in obese mice contributed to the subsequent decline in the number of NP-specific IFNγ+ CD8+ T cells observed at d8 p.i. Similar to our findings, a decrease in the total number of antigen-specific CD8+ T cells in the lungs of obese mice has been observed by others [17]. A new finding obtained from these experiments reveals that the CD8+ T cells in the BAL of obese mice were qualitatively different, in that TNFα response was elevated. The increased TNFα response of CD8+ T cells may have deleterious effects, as influenza virus-specific production of TNFα by CD8+ T cells has been shown to contribute to lung immunopathology, but not viral clearance [40]. At d11 p.i., lung lesions were assessed in our study with no difference in immunopathology observed. Perhaps viral/immune-mediated tissue damage was different between groups, but at a different time point than we assessed. In a separate study, at d14 p.i., inflammation remained present in the lungs of obese mice, but was no longer present in non-obese mice [15]. Increased antigen-specific CD8+ T cell derived TNFα may be one mechanism that contributes to the prolonged inflammation in the lungs of obese mice observed in other studies.

Our findings of reduced Type I IFN in the lungs of obese mice were consistent with other studies [13,14,17], and our results also showed that markers of IFN-inducible gene expression were reduced. At d3 p.i., IRF7 expression was increased ~ 60 fold in non-obese mice, but less than 10 fold in obese mice. Recognition of influenza virus by TLR7 in pDC leads to IFNα induction [45] and IRF7 may be induced by IFNα in a positive feedback fashion [46], leading to activation of a second, delayed IFNα set of genes including IFNα2 [47]. Both IRF7 and IFNα were significantly reduced in obese mice, suggesting a profound delay and/or decrease in this pathway. In unpublished findings, we have observed that lack of IFNα during the first several days of influenza viral infection resulted in delayed production of BAL cytokines and chemokines, and others have observed that IFNα pre-exposure to lung epithelial cells primes cells for greater chemokine production [44]. It is therefore possible that the early lack of IFNα in obese mice resulted in delayed cytokine or chemokine production. A deficiency of IFNα in obese mice during the first several days of infection may also have contributed to the reduced expansion of antigen-specific CD8+ T cells that we observed, and/or the development and maintenance of memory T cells observed in another study [19]. Type I IFN has been shown to be essential in the expansion of CD8+ T cells and in the development of memory T cells during viral infection [48,49,50]. Type I IFN produced during viral infection also promotes B cell antibody class switching [48,51,52]. The finding that exercised augmented anti-influenza virus-specific IgG2c may reflect the increased Type I IFN response that was observed at day 1 p.i. in non-obese mice and at day 3 p.i. in obese mice, although this possibility warrants further investigation. We have observed that IFNα is necessary during the first 4 days of infection for optimal antibody response (unpublished findings). Our findings, along with those from other published studies indicate that obesity impairs Type I interferon responses, and given the consistent findings across studies, further research into the deficiency of Type I interferon during obesity is warranted as this may prove to be therapeutically relevant for this population.

To our knowledge, these results are the first to demonstrate that the obese condition results in reduced tracheal ciliary beat frequency (CBF), a model for mucociliary clearance. The exact mechanism responsible for this finding remains to be identified, however, CBF is recognized as a mechanical innate immune defense as the mucociliary ‘escalator’ is active, even in the absence of infection, to clear inhaled foreign particles up and out of the lungs, and over into the esophagus [53]. It is possible that elevated concentrations of cytokines or chemokines (pre-infection) in obese mice contribute to the deficiency in CBF, as prior exposure to IL-8 has been shown to reduce isoproterenol-stimulated CBF [54]. As described in a recent review, several studies have observed elevated BAL cytokines or chemokines in obese mice [55]. However, these findings typically involve pulmonary inflammation models, and therefore it is less clear if obesity alone results in significantly elevated concentrations of inflammatory factors in the lung. It remains to be established as to whether the higher concentration of these cytokines/chemokines prior to infection mediates the obesity-related decrease in CBF.

A major objective of this study was to determine whether exercise treatment may attenuate obesity-related impairments of immunity to influenza virus, and establish the mechanisms responsible. In general the results demonstrated that exercise attenuated influenza-associated morbidity, and reversed many of the obesity related defects. It is relevant to note that although Ob EX mice had a slightly lower body fat percentage than Ob NO-EX mice, the metabolic parameters (serum leptin–S7 Fig) and total body weight of Ob EX mice were more characteristic of an obese mouse than that of non-obese mice. In this respect, the exercise-associated effects were not simply a result of restoring mice to a non-obese state. However, one limitation of the findings was that body fat was not exactly the same in exercised mice as compared to non-exercised mice, and it remains possible that this difference in body fat contributed to the observed differences of immune response. This type of limitation is common to exercise training studies and has been observed in other exercise studies with the high fat diet model [56]. Exercise treatment prior to infection minimized infection severity in terms of reduced weight loss in obese mice. In this infection model, weight loss typically reaches a peak on d7-9 p.i., and by this time point, obese mice had lost nearly 20% of body weight, whereas exercised obese mice lost slightly less than 15%. Although it may be expected that obese mice would exhibit greater morbidity that non-obese mice, weight loss may not be the optimal marker of morbidity during IAV infection in obese mice. Our results showed that a related measure of morbidity (kcal consumed per gram of body weight) began to increase more quickly after peak weight loss (day 8–9 p.i.) in non-obese mice compared to obese mice (S1 Fig); perhaps this reflects improved kinetics of illness recovery in non-obese mice that were not yet reflected in body weight data. Despite the improvement in symptom severity, viral load assessed by RT-PCR was not different in obese exercised mice during the first several days of infection. Surprisingly, the exercise-associated improvement in CBF did not result in reduced lung viral load as measured by PCR, yet it remains possible that the slight reduction in virus observed later was influenced by CBF. The modest reduction in viral load seen in Ob EX mice by day eight may have also been due to accelerated kinetics of viral clearance by CD8+ T cells. The total number of influenza virus NP-specific IFNγ+ CD8+ T cells was not greater in Ob EX mice relative to non-obese mice, but the cytokine profile differed in that CD8+ T cells from exercised mice produced more IFNγ and less TNFα. Greater IFNγ response to influenza promotes viral clearance and may be one pathway by which exercise may have improved the rate of viral clearance.

Based on findings demonstrating the diminished Type I IFN response in obese mice, we examined whether exercise would restore the IFN response. IFN-inducible gene expression of Ob EX mice were restored to levels nearly identical to that of non-obese mice. At d1 p.i., exercise treatment increased IFNα2 expression relative to non-exercised obese mice. IFNα2 is produced during the “second wave” of IFNα responses, following the early expression of IFNα4 [47]. Although IFNα4 was only increased 2-fold in exercised mice, the peak IFNα4 response likely occurred prior to 24 hours of infection. Perhaps the improvement in IFNα contributed to enhanced chemokine, cytokine, and cellular infiltration observed in exercised obese mice. Another early host defense pathway triggered by influenza viral infection is the inflammasome pathway involving NLRP3 and culminating in IL-1β and IL-18 activation and release. NLRP3 expression was only modestly increased by exercise, yet by d3 p.i., IL-1β concentration was greater in BAL of Ob EX compared to Ob NO-EX, raising the possibility that exercise may have enhanced inflammasome activation. At 24 hours p.i., a modest increase in the expression of chemokines was observed in Ob EX compared to Non-ob NO-EX, which was followed by ~ 2 fold increase in the BAL chemokine concentrations by day three. Along with an increase in chemokines exercise treatment partially restored the obesity-related decrease in BAL macrophages, pDC, TipDC, neutrophils, and monocytes. By day eight, Ob EX mice no longer showed a defect in the number of macrophages, neutrophils, dendritic cells, monocytes, or CD8+ T cells. An earlier activation of innate responses induced by exercise in obese mice may have contributed to the increased antibody elicited several days later. With respect to cytokines, on d3 p.i., exercise improved, but did not completely restore IL-1β, IL-6, TNFα, IL-12p70, IFNγ, IL-4 and IL-5. Using these data as a measure of T-helper type response, exercise did not appear to skew the response specifically towards or away from a Th1 response. Instead, both antibody response (IgG2c) and influenza virus-specific (NP-specific) CD8+ T cells were enhanced by exercise in obese mice. Overall, the results suggest that exercise improved immunity to influenza viral infection in obese mice and minimized symptom severity. In terms of specific mechanisms, it is possible that the earlier induction of IFNα resulted in increased expression of chemokines and cytokines, thus promoting leukocyte recruitment to the lungs and optimal activation of appropriate CD8+ T and antibody response. In future studies, it will be essential to confirm whether the early increase in IFNα is the major underlying mechanism responsible for exercise-induced improvement of immune response, and establish cellular pathways that contribute to this effect.

Exercise also reduced infection severity in non-obese mice, but the immune mechanisms appear to be quite different than those observed in obese mice. Exercise treatment again resulted in less weight loss during infection. However, in contrast to obese mice, a significant early reduction in lung viral load was found as a result of exercise treatment. One potential underlying mechanism contributing to the large decrease in viral load is a greater induction of interferon-inducible genes, particularly the interferon-inducible transmembrane protein (IFIT) genes observed 24 hours post-infection. In recent studies the IFIT family genes have been shown to possess potent antiviral effects against IAV infection, and are essential in limiting influenza virus-associated morbidity/mortality [57,58,59]. The significant early reduction in viral load likely resulted in reduced chemokine and cytokine expression as well as limited cell recruitment to the lungs. It has been shown that a higher dose of influenza virus is correlated with greater chemokine expression, cytokine expression, Type I IFN, and increased recruitment of innate immune cells to the lungs [60]. These same immune parameters were correlated with lung virus concentration in our study at day three, providing additional evidence that viral load predicts the magnitude of the subsequent immune response. It is important to note that although day three cellular recruitment, cytokine/chemokine activation were reduced by exercise in non-obese mice, the subsequent adaptive immune response was maintained or even enhanced by exercise. NP-specific CD8+T cells and IFNγ MFI were greater in exercise treated mice, and the CD8+ T cell percentage in the BAL was greater at d8 p.i. Anti-influenza virus-specific antibody response was also greater in exercised mice. A difference in lesion score was not found, although in previous studies we have observed that exercise treatment minimized influenza-associated immunopathology [26].

In summary, the results show promise not only in the use of exercise as a preventive measure to improve host defense against influenza viral infection, but provide a better understanding of mechanisms that may be essential in host protection from infection in the obese and non-obese state. It appears that Type I IFN responsiveness may be a key factor underlying the protective benefits of exercise. In non-obese mice, IFN-inducible gene expression may reduce viral load earlier during the course of infection, whereas in obese mice, exercise may restore the obesity-associated impairment in Type I IFN activation providing for appropriate IFNα-related immunomodulatory responses.

## Supporting Information

S1 Fig Morbidity is attenuated in the obese and non-obese exercised mice as indicated by increased kcal per gram body weight food intake over the course of an influenza viral infection. (TIF)

S2 Fig BAL viral load and lung viral load are highly correlated with one another. (TIF)

S3 Fig Lung viral load correlates with BAL cell numbers at day 3 and day 8 post-infection. (TIF)

S4 Fig Percentage of CD8+ T cells recruited after influenza viral infection correlates with BAL viral load in non-obese exercised mice. (TIF)

S5 Fig Percentage of macrophages recruited after influenza viral infection correlates with BAL viral load in non-obese exercised mice. (TIF)

S6 Fig Correlations between BAL viral load and levels of various chemokines were determined in non-obese mice at day 3 post-infection. (TIF)

S7 Fig Serum leptin concentration is altered by obesity. (TIF)

S1 Table Cytokines and chemokines (pg/mL) in BAL at day three and eight post-influenza infection. (DOC)

S2 Table BAL cytokine and chemokine detected at baseline in non-infected obese and non-obese mice. (DOCX)

S1 Video Ciliary beat in a tracheal ring from a male C57BL/6 mice. (AVI)
