# Supplementary material for: Exercise Improves Host Response to Influenza Viral Infection in Obese and Non-Obese Mice through Different Mechanisms
Source: PLoS One. 2015 Jun 25;10(6):e0129713. doi: 10.1371/journal.pone.0129713 (PMC4482026; doi:10.1371/journal.pone.0129713)
Supplement: S1 Table — (DOC) [file pone.0129713.s008.doc]

| **Protein**  **S1 Table. Cytokines and chemokines (pg/ml) in BAL at day three and eight post-influenza infection.**  **(Day3/8p.i)** | **Non-obese No-Ex (Mean±SEM)** | **Non-obese--Ex**  **(Mean±SEM)** | **Obese-No-Ex**  **(Mean±SEM)** | **Obese-Ex**  **(Mean±SEM)** | **p-value** |
| --- | --- | --- | --- | --- | --- |
| **GM-CSF d3**  **d8** | 63.5+11.8  46.1+5.7 | 19.9+7.6  43.3+3.5 | 8.2+5.3  36.3+6.3 | 15.6+5.9  44.7+2.8 | d*,e*,d*e |
| **KC d3**  **(CXCL1) d8** | 752.4+121.7  151.3+26.5 | 236.4+88.1  172.6+23.1 | 109.4+63.1  146.2+21 | 188.0+69.3  144.3+13.4 | d*,e*,d*e |
| **MIG d3**  **(CXCL9) d8** | 13020.1+1628.8  13129.7+2762.9 | 3498.7±1371.3  9530.1+2078.7 | 1380.4±1041.6  14731.4+2791.5 | 3214±1177.6  11934.3+3075 | d*,e*, d*e |
| **MIP-1β d3**  **(CCL4) d8** | 280.5±49.4  278.6+66.1 | 77.8±32.8  157.5+28.1 | 37.9±23.1  212.8+41.6 | 82.8±21.2  198.4+28.9 | d*,e*, d*e  e+, |
| **MIP-2 d3**  **(CXCL2) d8** | 267.9±26.5  88.1+14.8 | 129.0±27.2  63.1+8.6 | 62.4±29.3  67.1+9.0 | 120.7+24.6  74.5+9.5 | d*, e+,d*e |
| **MIP-3α d3**  **(CCL20)** | 122.8±14.5 | 121.6±59.9 | 32.8±10.8 | 75.2±17.1 | d+ |
| **MIP-3β d3**  **(CCL19)** | 96.1±11.7 | 20.7±13.5 | 10.4±9.2 | 49±14.6 | d+, d*e |
| **MCP-5 d3**  **(CCL12)** | 695.3±98.4 | 260.8±104.2 | 68.3±36.6 | 236.71±64.6 | d*, d*e |
