# Supplementary material for: Exercise Improves Host Response to Influenza Viral Infection in Obese and Non-Obese Mice through Different Mechanisms
Source: PLoS One. 2015 Jun 25;10(6):e0129713. doi: 10.1371/journal.pone.0129713 (PMC4482026; doi:10.1371/journal.pone.0129713)
Supplement: S2 Table — (DOCX) [file pone.0129713.s009.docx]

| **Protein**  **S2 Table. BAL cytokine and chemokine detected at baseline in non-infected obese and non-obese mice.** | **Non-obese No-Ex**  **Non-infected**  **(Mean±SEM)** | **Obese No-Ex**  **Non-infected**  **(Mean±SEM)** |
| --- | --- | --- |
| **GM-CSF** | not detectable | not detectable |
| **KC**  **(CXCL1)** | 1.75 ± 0.448 | 2.33 ± 0.265 |
| **MIG**  **(CXCL9)** | 4.606 ± 2.646 | 3.61 ± 2.12 |
| **MIP-1β**  **(CCL4)** | not detectable | 2.99 ± 1.784 |
| **MIP-2**  **(CXCL2)** | not detectable | not detectable |
| **MIP-3α**  **(CXCL2)** | not detectable | not measured |
| **MIP-3β**  **(CXCL2)** | not detectable | not measured |
| **CCL12**  **(MCP-5)** | not detectable | not measured |
